# Calmodulin Interactions with Voltage-Gated Sodium Channels

**DOI:** 10.3390/ijms22189798

**Published:** 2021-09-10

**Authors:** Xin Wu, Liang Hong

**Affiliations:** Department of Medicine, University of Illinois at Chicago, Chicago, IL 60612, USA; xinwu@uic.edu

**Keywords:** calmodulin, voltage-gated sodium channel, IQ domain, sodium channel isoform, genetic mutation

## Abstract

Calmodulin (CaM) is a small protein that acts as a ubiquitous signal transducer and regulates neuronal plasticity, muscle contraction, and immune response. It interacts with ion channels and plays regulatory roles in cellular electrophysiology. CaM modulates the voltage-gated sodium channel gating process, alters sodium current density, and regulates sodium channel protein trafficking and expression. Many mutations in the CaM-binding IQ domain give rise to diseases including epilepsy, autism, and arrhythmias by interfering with CaM interaction with the channel. In the present review, we discuss CaM interactions with the voltage-gated sodium channel and modulators involved in CaM regulation, as well as summarize CaM-binding IQ domain mutations associated with human diseases in the voltage-gated sodium channel family.

## 1. Introduction

**Figure 1:**
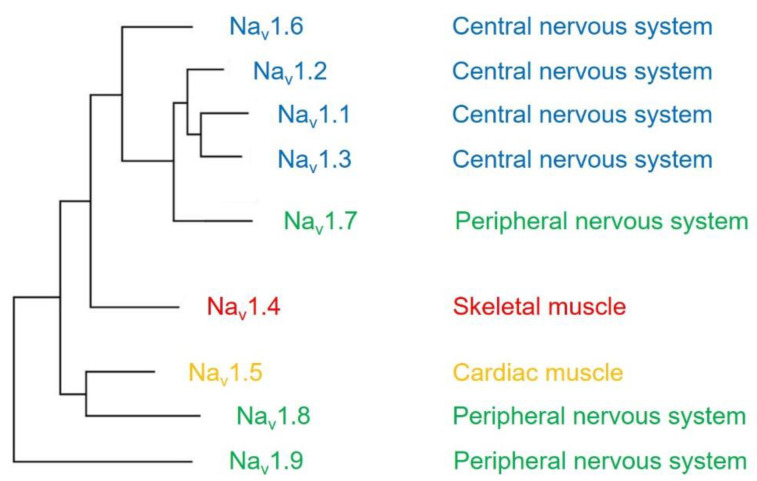
A phylogenetic tree and tissue distribution of voltage-gated sodium channels. Isoforms predominantly expressed in the central nervous system (blue), peripheral nervous system (green), skeletal muscle (red), and cardiac muscle (orange) are highlighted.

The voltage-gated sodium channel (Na_v_) plays a vital role in the generation and propagation of action potential in excitable cells such as neurons and cardiac myocytes [1]. The family of voltage-gated sodium channels has nine members named Na_v_1.1 through Na_v_1.9 [2]. Among them, Na_v_1.1, Na_v_1.2, Na_v_1.3, and Na_v_1.6 are predominantly expressed in neurons of the central nervous system [3,4]. Three isoforms (Na_v_1.7, Na_v_1.8, and Na_v_1.9) are widely expressed in neurons of the peripheral nervous system, such as dorsal root ganglia (DRG) neurons [5,6,7]. The isoform Na_v_1.4 is responsible for upstroke of the action potential in skeletal muscle [8], and Na_v_1.5 is a cardiac-specific isoform known as cardiac sodium channel (Figure 1) [9].

An essential property of voltage-gated sodium channels is “inactivation”, which prevents the reopening of the channel until complete recovery [10]. The inactivation regulates the frequency of action potential firing initiated by sodium channels in excitable cells. It reduces the breakdown of ionic gradients and cell death.

All voltage-gated sodium channels contain a calmodulin (CaM)-binding IQ domain necessary for the channel inactivation [11]. CaM is a small protein expressed in all eukaryotic cells [12]. It acts as a ubiquitous signal transducer and regulates essential processes such as neuronal plasticity, muscle contraction, and immune response [13]. CaM has two globular domains (i.e., N-terminal lobe and C-terminal lobe) connected by a linker. Each lobe contains two EF-hand motifs binding to Ca^2+^ [14]. In the Ca^2+^-free state, the EF-hands are collapsed in a compact configuration. When Ca^2+^ is bound to CaM, a conformational change occurs in the protein and rearranges the structural information of CaM. 

The CaM-binding IQ domain is located within the C-terminal domain of the voltage-gated sodium channel. It contains around 25 residues with two highly conserved amino acids, isoleucine (I) and glutamine (Q), in the middle of the motif. The residues in the IQ domain form a seven-turn α-helix binding to CaM in a Ca^2+^-independent manner. In addition to the IQ domain, a globular domain in the C-terminus of voltage-gated sodium channel consists of EF hand-like (EFL) motifs that interact with CaM in the absence of Ca^2+^ [15,16], whereas an intracellular loop connecting domains III and IV (III-IV linker) in some isoforms of the sodium channel is shown to interact with CaM in the presence of Ca^2+^ [17]. 

## 2. CaM Regulation of Voltage-Gated Sodium Channel

CaM binds to the voltage-gated sodium channel and modulates the sodium channel gating process [15], alters sodium current density [18], and regulates sodium channel protein trafficking and expression [19].

**Figure 2:**
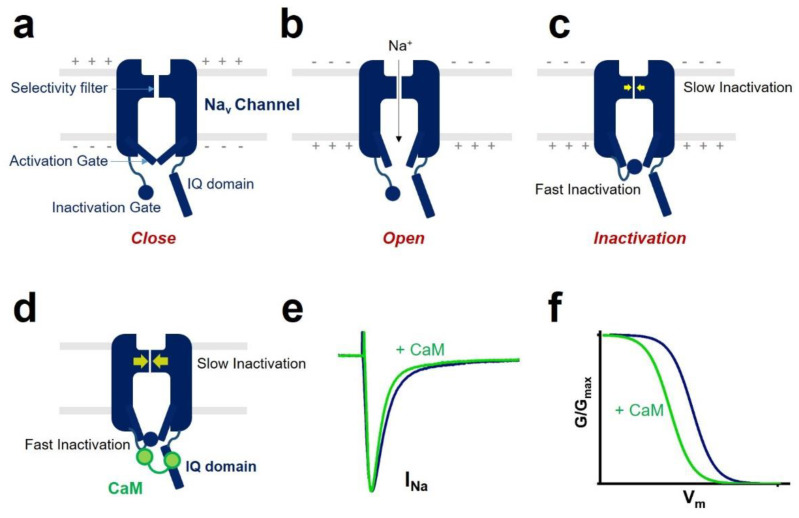
CaM regulation of sodium channel function by interaction with the IQ domain. (**a-c**) Voltage-gated sodium channel (Na_v_) has three states known as “close” (**a**), “open” (**b**), and “inactivation” (**c**). Several functional parts (inactivation gate, activation gate, and selectivity filter) are involved in channel gating. The “+”and ”-” represent charge separation across the membrane. Differences in charges on opposite sides of the cellular membrane generate the membrane potential. At rest, the activation gate is closed (**a**). When the membrane is depolarized, the activation gate opens and allows Na^+^ to enter (**b**). At the same time, depolarization of the membrane causes the inactivation gate to close and induces the fast inactivation. The channel also shows slow inactivation related to conformational rearrangements of the selectivity filter (highlighted in yellow arrows in **c**). (**d**–**f**) CaM binds to the voltage-gated sodium channel IQ domain and modulates the sodium channel inactivation. CaM affects channel’s fast inactivation kinetics (**e**) by an interaction between the inactivation gate formed by cytoplasmic III-IV linker and the C-terminal domain of the channel (**d**). CaM enhances the slow inactivation (highlighted in bigger yellow arrows in **d**). It induces a hyperpolarized shift of voltage dependence of inactivation and reduces the channel’s availability (**f**).

One of the CaM modulations of voltage-gated sodium channel gating is inactivation (Figure 2). Voltage-gated sodium channel has two different types of inactivation, known as fast inactivation and slow inactivation. Fast inactivation occurs by occlusion of the intracellular pore by an “inactivation gate” formed by the cytoplasmic III-IV linker [10,20]. Slow inactivation is related to conformational rearrangements of the selectivity filter of the channel [21,22]. CaM mediates the fast inactivation by an interaction between III-IV linker and C-terminal domain of the voltage-gated sodium channel [23]. It enhances the slow inactivation by inducing a hyperpolarized shift of voltage dependence of inactivation and reducing the channel’s availability. CaM interaction with the sodium channel affects channel inactivation kinetics. It strengthens the development of inactivation and slows the recovery from inactivated state of the sodium channel. 

In some isoforms of the voltage-gated sodium channels, CaM influences the activation process. It was reported that co-expression of CaM and Na_v_1.1 produced a hyperpolarized shift of the voltage dependence of activation of the Na_v_1.1 sodium channel [24]. 

Moreover, CaM can modulate sodium channel current density [18]. Studies reported that CaM regulation of sodium current density is Ca^2+^-dependent [24,25]. CaM does not affect sodium current amplitude in the absence of Ca^2+^. In contrast, it increases the current density in the presence of a high concentration of Ca^2+^. 

In addition to modulation of the gating process of the voltage-gated sodium channel, CaM influences sodium channel protein trafficking and expression. It was shown to mediate the cell surface expression of Na_v_1.4 protein through the interaction with the IQ domain of the sodium channel [19].

## 3. Modulators Involved in CaM Regulation of Sodium Channel

There are several cellular partners involved in CaM regulation of the voltage-gated sodium channel function such as FHF (fibroblast growth factor homologous factor) [26]. CaMKII (calcium/calmodulin-dependent protein kinase II) [27], and cations (Ca^2+^, Mg^2+^) [11,28,29].

**Figure 3:**
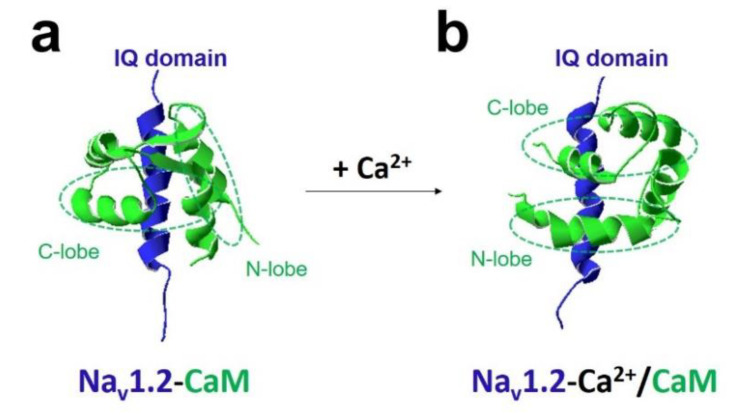
Ca^2+^ modulates the interaction between CaM and Na_v_1.2 IQ domain. The NMR structures showed Na_v_1.2 IQ domain (blue) in complex with CaM (green) in the absence (**a**) or presence (**b**) of Ca^2+^. Two lobes (N-lobe and C-lobe) of CaM are indicated. C-lobe of CaM bound to the Na_v_1.2 channel IQ domain independent of Ca^2+^ concentration. In the absence of Ca^2+^, the N-lobe of CaM did not interact with the IQ domain (PDB: 2KXW) (**a**). When binding to Ca^2+^, the N-lobe of CaM was induced to interact with the distal IQ domain of Na_v_1.2 channel (PDB: 2M5E) (**b**).

**Figure 4:**
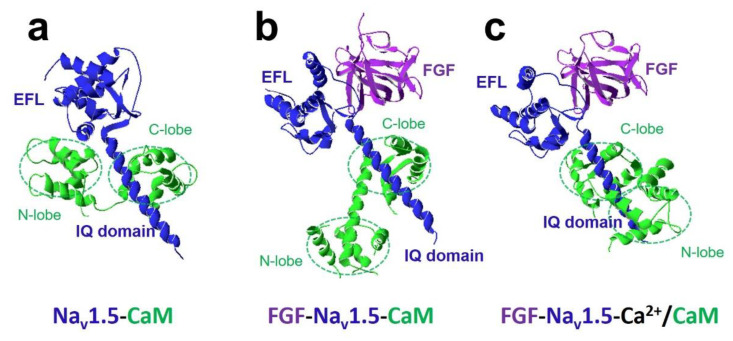
FGF and Ca^2+^ modulate the interaction between CaM and Na_v_1.5 IQ domain. (**a**) The structure of the complex of the C-terminal domain of Na_v_1.5 with CaM showed C-lobe of CaM bound to the IQ domain, and N-lobe of CaM interacted with EFL (PDB: 4OVN). (**b**) Crystal structure showed the interactions between the C-terminal domain of Na_v_1.5, FGF, and CaM (PDB: 4DCK). When FGF bound to the channel, the N-lobe of CaM was rearranged. It did not make contact with EFL and was free to interact with other parts of the channel complex. The N-lobe of CaM likely interacted with the inactivation gate of the sodium channel and modulated the channel’s inactivation. (**c**) In a high concentration of Ca^2+^, the N-lobe of CaM was induced to interact with the distal IQ domain of Na_v_1.5 channel (PDB: 4JQ0).

The most crucial regulator is Ca^2+^ [11,28]. In the presence of Ca^2+^, Ca^2+^/CaM (Ca^2+^-binding form of CaM) has a different regulation of sodium channel function compared with apo-CaM (Ca^2+^ free CaM), and Ca^2+^ binding to CaM induces rearrangements between the voltage-gated sodium channel and CaM lobes. In the absence of Ca^2+^, the N-lobe of CaM does not target the IQ domain (Figure 3a and Figure 4a,b). In the presence of Ca^2+^, the structure of the N-lobe is rearranged by Ca^2+^. It generates an allosterically conformational change binding to the distal IQ domain of the channel (Figure 3b and Figure 4c). However, the C-lobe of CaM can bind to the voltage-gated sodium channel IQ domain in a Ca^2+^-independent manner. 

The FHFs were shown to form a complex with CaM and modulate CaM regulation of Na_v_1.5 function [26]. A recent study discovered fibroblast growth factor homologous factor FGF13 (fibroblast growth factor 13, also known as FHF2) tuned arrhythmogenic late sodium currents in CaM binding-deficient channels in cardiac myocytes [26]. The crystal structures in Figure 4 showed that FGF and Ca^2+^ mediated the interaction between CaM and Na_v_1.5 IQ domain. It indicated that the N-lobe of CaM interacted with the EFL of the channel in the absence of FGF. However, when FGF was bound to the channel, the N-lobe of CaM was rearranged. It did not make contact with the EFL and was free to interact with other parts of the channel complex. The N-lobe of CaM likely interacted with the III-IV linker of the sodium channel and modulated the channel’s inactivation.

In addition, CaMKII has been reported to influence CaM interaction with the sodium channel. One study found CaMKII-mediated phosphorylation of the Na_v_1.1 sodium channel IQ domain increased CaM binding affinity to the channel [27]. Three phosphorylation sites (T1909, S1918, and T1934) were identified in the Na_v_1.1 IQ domain. Other studies demonstrated that CaMKII phosphorylated Na_v_1.5 at residue S571 to decrease channel availability. The CaMKII phosphorylation site S571 is located at the intracellular DI-DII loop of the Na_v_1.5 sodium channel, and mutation at position S571 abolished the hyperpolarized shift of the voltage dependence of inactivation produced by CaMKII phosphorylation. The effects of CaMKII-mediated phosphorylation of IQ domain on sodium currents have not been characterized. CaMKII-dependent IQ domain phosphorylation might confer normal sodium currents important for the function of cells where the Na_v_ channels are expressed. The CaMKII-mediated effect seems to be isoform-specific. CaMKII phosphorylation enhances CaM affinity for sodium channel Na_v_1.1 but not for Na_v_1.2 [27,30]. 

Moreover, Mg^2+^ is involved in the CaM-mediated modulation. In some neurons, low Mg^2+^ treatment increases the effects of CaM on the voltage-gated sodium channel activity [29].

With these partners, CaM regulates voltage-gated sodium channel function by altering channel gating, sodium current density, and expression of the sodium channel protein. Studies have shown that CaM has isoform-specific modulation of voltage-gated sodium channel function [31,32,33,34].

## 4. Interaction between CaM and Sodium Channel Isoforms

### 4.1. Na_v_1.1 

Na_v_1.1 is one of the voltage-gated sodium channels predominantly expressed in the central nervous system [35]. It is known to be highly expressed in the soma and apical dendrite of the Purkinje cells.

The effect of CaM on Na_v_1.1 channel function is Ca^2+^-dependent. CaM does not affect sodium current density of wild-type Na_v_1.1 channel in the absence of Ca^2+^ [25]. but significantly increases Na_v_1.1 current density in the presence of 10µM intracellular Ca^2+^ [24]. 

CaM mediates Na_v_1.1 channel gating process when binding to the IQ domain, and overexpression of CaM induces a hyperpolarized shift of the voltage dependence of activation of Na_v_1.1 [24]. However, it does not generate significant effects on the voltage dependence of inactivation at high intracellular Ca^2+^ concentration but accelerates inactivation process of Na_v_1.1 with low Ca^2+^ [24]. 

A GST pull-down assay reported CaM interaction with Na_v_1.1 sodium channel IQ domain [27]. The Na_v_1.1 IQ domain preferentially binds to apoCaM than Ca^2+^/CaM. In the absence of Ca^2+^, C-lobe of CaM is the predominant domain binding to Na_v_1.1, and N-lobe of CaM is proposed to interact with other parts of the channel [36]. In the presence of Ca^2+^, N-lobe of CaM is shown to be the predominant domain binding to Na_v_1.1 [27]. Moreover, one study reported that CaMKII-mediated phosphorylation of Na_v_1.1 enhanced CaM interaction with IQ domain of the Na_v_1.1 channel [27].

### 4.2. Na_v_1.2 

The neuronal sodium channel Na_v_1.2 is expressed in granule cells and interneurons in the central nervous system [37], and plays roles in excitatory neurons in the neocortex and hippocampus [38].

CaM has been shown to mediate Ca^2+^-dependent regulation of the Na_v_1.2 channel [39]. CaM binding to Na_v_1.2 reduces Ca^2+^ binding affinity in the CaM-binding sites [40]. Although there is a Ca^2+^-binding EFL motif upstream of the IQ domain in the Na_v_1.2 channel, the Ca^2+^-mediated changes in modification of Na_v_1.2 function are proposed to be likely from the interaction between CaM and Na_v_1.2 IQ domain when Ca^2+^ binding to CaM. The Na_v_1.2 channel EFL motif was indicated to enhance Ca^2+^ binding to CaM interacting with the channel [41].

Structural study analysis showed the C-lobe of CaM anchored to the Na_v_1.2 channel IQ domain independent of Ca^2+^ concentration. In the absence of Ca^2+^, the N-lobe of CaM did not interact with the IQ domain. When binding to Ca^2+^, the N-lobe of CaM was induced to interact with the distal IQ domain of the Na_v_1.2 channel (Figure 3) [42].

CaM has no notable effects on the Na_v_1.2 current density. Overexpression of CaM does not significantly decrease the peak sodium current amplitude of the Na_v_1.2 channel [43]. 

### 4.3. Na_v_1.3 

Although Na_v_1.3 is a neuronal sodium channel [44], the first paper reporting CaM regulation of Na_v_1.3 is from a study on microvessels of the kidney [45]. 

The study examined the expression of Na_v_1.3 in rat descending vasa recta, a series of blood vessels that perfuse the renal medulla. Further, it verified that CaM binding to the C-terminus of Na_v_1.3 by pull-down and immunoprecipitation assays [45]. The voltage-gated sodium currents in the vasa recta pericytes were remarkably suppressed by CaM inhibitors CIP (calmodulin inhibitory peptide) and W7 (N-(6-aminohexyl)-5-chloro-1-naphthalene-sulphonamide hydrochloride). However, CIP or W7 did not generate alteration of gating process of endogenous voltage-gated sodium currents in vasa recta cells [45]. 

Another study in hippocampal neurons showed that CaM regulates Na_v_ channel function in an Mg^2+^-dependent manner. The Na_v_1.3 activities, together with Na_v_1.1 and Na_v_1.2, were more sensitive to Ca^2+^/CaM regulation in a low-Mg^2+^ environment than normal neurons [29].

### 4.4. Na_v_1.4 

Na_v_1.4 is the predominant subtype of sodium channel initiating skeletal muscle action potential [8]. Na_v_1.4 mutations associated with periodic paralysis disorders affect skeletal muscle excitability [46]. 

CaM influences the gating process of the Na_v_1.4 sodium channel, and the effects of CaM on Na_v_1.4 inactivation are related to Ca^2+^ [31]. In the presence of Ca^2+^, CaM shifts the voltage dependence of inactivation of the channel to hyperpolarizing direction. When the free Ca^2+^ is removed, the CaM-induced shift of the steady-state inactivation curve is attenuated [31]. 

CaM regulates Na_v_1.4 sodium current density. The decreased sodium currents caused by Na_v_1.4 IQ mutations were shown to be rescued by overexpression of CaM. However, CaM does not significantly affect the sodium current density of the wild-type Na_v_1.4 channel [32].

In addition to functional regulation, CaM influences Na_v_1.4 channel trafficking and expression. One study demonstrated an intimate relationship between intact Na_v_1.4 channels and CaM in live cells and revealed that CaM participated in the regulation of cell surface expression of Na_v_1.4 protein through the interaction with the Na_v_1.4 channel IQ domain [19].

### 4.5. Na_v_1.5 

The most well-studied voltage-gated sodium channel interacting with CaM is the Na_v_1.5. The cardiac sodium channel Na_v_1.5, encoded by the *SCN5A* gene, plays a critical role in the fast depolarization of the cardiac action potential [47]. Cardiac sodium channel dysfunction caused by Na_v_1.5 mutations was reported to remodel abnormal action potential underlying arrhythmias [48].

CaM binds to Na_v_1.5 C-terminal domain and enhances the inactivation of Na_v_1.5 channel. It shifts the steady-state inactivation to hyperpolarization and influences inactivation kinetic [49]. CaM mediates Ca^2+^ regulation of Na_v_1.5 sodium channel function [50]. In the presence of Ca^2+^, CaM is promoted to bind to the Na_v_1.5 inactivation gate. Perturbation of the interaction between the gate and CaM generates a decreased recovery from inactivation of the Na_v_1.5 channel [51,52]. 

The sodium channel Na_v_1.5 IQ domain mutations associated with arrhythmia reduce CaM binding affinity. In HEK293 cells, overexpression of CaM attenuates late sodium currents caused by Na_v_1.5 IQ domain mutations [43]. CaM binding to Na_v_1.5 channel IQ domain also modifies late sodium currents in cardiac myocytes [26]. Study in transgenic mouse models showed FHFs tuned cardiac late sodium currents in ventricular myocytes. FHFs diminished late sodium current of the Na_v_1.5 channel IQ/AA mutation (substitution of two conserved residues isoleucine (I) and glutamine (Q) with double alanines (AA) in the IQ domain), IQ/AA mutation has been reported to disrupt CaM binding to the IQ domain of voltage-gated sodium channels. 

Studies in guinea-pig ventricular myocytes revealed CaM enhances the cardiac sodium current density [53]; this is different from the result that CaM does not influence on sodium current amplitude when co-expressing with Na_v_1.5 channel in HEK293 cells [43]. It indicates that some environmental factors in cardiac tissues are involved in CaM regulation of Na_v_ function. 

Additionally, a recent study proposed CaM can bind to the N-terminal domain of the cardiac Na_v_1.5 channel, wherein one Brugada syndrome-associated mutation in the N-terminal domain of Na_v_1.5 was shown to weaken the interaction between CaM and Na_v_1.5 N-terminal domain [54].

### 4.6. Na_v_1.6 

Na_v_1.6 is the primary voltage-gated sodium channel at the myelin-sheath gaps. It is important in the generation and propagation of the action potential along myelinated axons [55]. 

CaM binding to Na_v_1.6 is crucial for functional sodium current expression [32]. Disruption of CaM binding to the Na_v_1.6 channel significantly reduces sodium current amplitude. Overexpression of CaM rescues the decreased sodium currents caused by Na_v_1.6 channel CaM-binding domain mutations. CaM enhances the rate of Na_v_1.6 inactivation and switches Na_v_1.6 channel from fast mode to slow mode. It has no effects on the voltage dependence of activation of Na_v_1.6 [32]. 

The structural study reported that CaM interacted with different residues of the Na_v_1.6 channel, depending on the absence or presence of Ca^2+^. However, three key residues (Arg1902, Tyr1904, and Arg1905) in the Na_v_1.6 channel IQ domain were identified to interact with CaM in a Ca^2+^-independent manner [56].

### 4.7. Na_v_1.7 

The voltage-gated sodium channel Na_v_1.7 is highly expressed in nociceptive dorsal root ganglion (DRG) neurons and superior cervical ganglion (SCG) neurons [57]. 

The electrophysiological study on CaM interacting with Na_v_1.7 has not yet been determined. One nuclear magnetic resonance (NMR) study analyzed complexes of calcium-free CaM bound to peptides of IQ motifs of Na_v_1.7. It showed C-lobe of CaM contributed to the interface with the IQ motif of Na_v_1.7 and the N-lobe of CaM interacted with other parts of the sodium channel [36]. These results are consistent with findings in other voltage-gated sodium channels and suggest that an interaction interface between CaM and IQ domain of voltage-gated sodium channels is highly conserved in the C-lobe of CaM.

### 4.8. Na_v_1.8 

Na_v_1.8 channel is expressed in dorsal root ganglia (DRG) neurons [6], and genetic research has shown this channel is also implicated in cardiac function [58], in which Na_v_1.8 was identified in cardiomyocytes and intracardiac neurons, contributing to cardiac repolarization [59].

CaM is a functional partner of Na_v_1.8 sodium and interacts with Na_v_1.8 in vivo [18]. It was found that CaM can coimmunoprecipitate with endogenous Na_v_1.8 channels from native DRG. CaM mediated a frequency-dependent inhibition of sodium channels in the DRG neurons. The sodium currents in neurons were significantly decreased in the presence of high cellular Ca^2+^ (10 µM free Ca^2+^) and CaM antagonist calmodulin-binding peptide (CBP), suggesting CaM modulated Na_v_1.8 current density in neurons [18]. 

A recent study demonstrated the CaM enhanced slow inactivation of the Na_v_1.8 channel and reduced channel availability. It modulated Na_v_1.8 function through its interaction with the IQ domain. CaM mediated regulation was abrogated in mutations disrupting CaM binding to the channel [60]. 

### 4.9. Na_v_1.9 

Na_v_1.9 is expressed in sensory neurons of the DRG and trigeminal ganglion [61]. 

**Figure 5:**
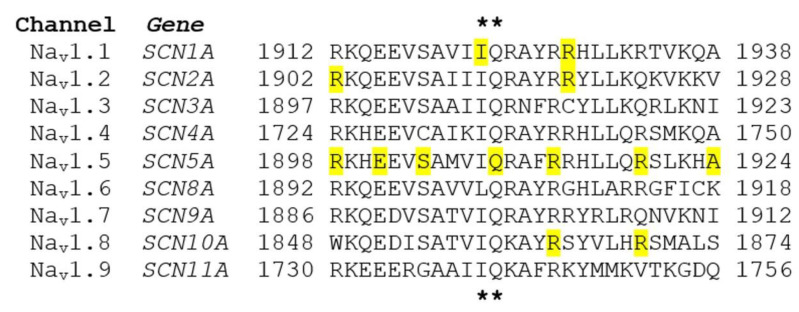
Alignment of voltage-gated sodium channel family IQ domain and CaM-binding IQ domain mutations. Sodium channel mutations in the IQ domain associated with human diseases are highlighted in yellow. The conserved residues Ile (I) and Glu (Q) are indicated by stars.

To date, there is no study investigating the interaction between CaM and Na_v_1.9 channel. The Na_v_1.9 IQ domain shares high sequence homology with other sodium channels (Figure 5). It remains to be determined if CaM regulation of Na_v_1.9 channel plays a role in channel function in sensory neurons.

## 5. CaM-Binding IQ Domain Mutations in Voltage-Gated Sodium Channel

A large number of voltage-gated sodium channel mutations have been linked with disorders of the nervous and cardiovascular systems [62]. Some mutations are located in the highly conserved CaM-binding IQ domain and cause severe neurological diseases and cardiac arrhythmia (Figure 5, Table 1). 

### 5.1. Na_v_1.1 Mutations 

Na_v_1.1 mutations are associated with familial epilepsies. The Na_v_1.1-I1922T was identified in patients with Dravet syndrome [63,64]. In this mutation, highly conserved residue isoleucine (I) that plays a critical role in CaM binding to the IQ domain was substituted by threonine (T). Another mutation, Na_v_1.1-R1928G, is linked with cryptogenic epileptic syndrome [65], and characterized in patients diagnosed with generalized epilepsy with febrile seizures plus (GEFS+) [66], familial hemiplegic migraine (FHM) [67], and severe myoclonic epilepsy of infancy (SMEI) [68]. 

Additionally, CaM was reported to generate partially functional rescue of Na_v_1.1-M1841T, an epileptogenic mutation located upstream of the CaM-binding IQ domain of the Na_v_1.1 channel [25]. M1841T was characterized as a loss-of-function mutant and generated very small sodium currents. Co-expression of CaM with M1841T partially rescued the loss of function of the channel and induced a 3.5-fold increase in sodium current amplitude [25].

### 5.2. Na_v_1.2 Mutations 

The Na_v_1.2 mutation R1902C has been identified in patients with familial autism [69]. Na_v_1.2-R1902C perturbed Ca^2+^-dependent changes in the regulation of Na_v_1.2 function [39]. Protein pull-down assays showed R1902C abolished Ca^2+^-dependence of CaM binding [30]. In the presence of Ca^2+^, Na_v_1.2-R1902C induced a hyperpolarization shift of the voltage dependence of activation and inactivation of the channel [42].

The mutation Na_v_1.2-R1918H was associated with idiopathic generalized epilepsy [70]. This mutation produced a pathogenically increased late sodium current, indicating a gain-of-function mechanism in Na_v_1.2 mutations associated with epilepsy. The enhanced late sodium current mediated by Na_v_1.2-R1918H was able to be rescued by CaM overexpression [43].

### 5.3. Na_v_1.5 Mutations 

The Na_v_1.5-R1898H is a mutation located at the N-terminal end of the CaM-binding IQ domain of the Na_v_1.5 channel and was characterized in a patient with arrhythmogenic right ventricular dysplasia/cardiomyopathy (AVRD/C) [71]. A study assessed this mutation’s cellular and molecular phenotype using an induced pluripotent stem cell-derived cardiomyocytes (iPSC-CMs) approach. It showed that R1898H was a loss-of-function mutation that caused around 40% reduction in peak sodium current [71]. Three-dimensional super-resolution fluorescence microscopy (3D-SRFM) experiments showed a structural deficit of Na_v_1.5 and N-cadherin clusters in the R1898H mutation cells, indicating a role that Na_v_1.5 dysfunction associated with R1898H mediated cardiomyopathy [71]. Another mutation at the same position, R1898 (Na_v_1.5-R1898C), was identified in a patient with Brugada syndrome [72]. It should be noted that the Na_v_1.5-R1898C corresponds to the autism mutation Na_v_1.2-R1902C (Figure 5). In Na_v_1.2, the mutation (Na_v_1.2-R1902C) affected the gating process of the channel and perturbed Ca^2+^-dependent regulation of Na_v_1.2 function [42]. It is likely that the same mutation in the Na_v_1.5 (Na_v_1.5-R1898C) generated similar consequences on the Na_v_1.5 function and played similar roles in cardiac diseases.

There are two mutations at the position Na_v_1.5-E1901 [73,74]. The Na_v_1.5-E1901K was associated with Brugada syndrome [74], and Na_v_1.5-E1901Q was identified in a patient with type 3 of the long-QT syndrome (LQT3). Na_v_1.5-E1901Q was shown to cause an increased late sodium current [73]. In the absence of Ca^2+^, the negative residue Glu at the position 1901 (E1901) might couple with a positive residue of CaM to form charge–charge interaction, stabilizing CaM binding to the IQ domain of the channel [75]. Replacement of Glu with Gln (E1901Q) or Lys (E1901K) likely disrupted the salt bridge formed by the charge–charge interaction between CaM and Na_v_1.5 IQ domain and produced a perturbation of CaM regulation of the channel, providing an explanation for the pathological roles of those mutations in Brugada syndrome and LQT3 syndrome.

The Na_v_1.5 mutation S1904L was associated with Brugada and long-QT syndromes [74,76]. The position of Ser1904 was proposed to use hydrogen bonds to interact with the main chain of the C-lobe of the CaM. Mutation at the position of 1904 (S1904L) perturbed the Na_v_1.5 channel interaction with CaM. In the absence of Ca^2+^, a flow cytometric FRET two-hybrid analysis between the S1904L and CaM demonstrated a weaker affinity compared to the wild-type Na_v_1.5 channel [15]. Additionally, S1904L induced an enhanced late sodium current, indicating a delayed inactivation of the channel caused by CaM dysregulation of the channel in this mutation [76].

Na_v_1.5-Q1909R was linked with sudden infant death syndrome (SIDS) and LQT3 syndrome [77,78]. Functional studies showed that Q1909R is a gain-of-function mutation [79]. Ventricular action potential (AP) simulations showed a frequency-dependent reduction of AP duration in Q1909R carriers [79]. Moreover, Q1909R resulted in an increase in late sodium current. Co-expression of CaM or higher intracellular Ca^2+^ reduced the enhanced late sodium currents [43,79].

The mutation Na_v_1.5-R1913H was identified in a patient with LQT3 syndrome [80]. In the absence of Ca^2+^, the position Arg1913 interacts with negative residues of C-lobe of CaM. Mutation at this position (R1913H) resulted in an enhanced late sodium current, and overexpression of CaM can reduce the enhanced late sodium current [43]. Another mutation (R1919C) was reported to be associated with Brugada syndrome and Long-QT syndrome [81]. 

A Brugada syndrome mutation A1924T is located at the C-terminal end of the IQ domain. Na_v_1.5-A1924T induced a negative shift in voltage-state activation, which would cause some persistent depolarization of the channel [82]. Na_v_1.5-A1924T showed a reduced slow inactivation, and application of CaM rescued the decreased slow inactivation caused by Na_v_1.5-A1924T [49]. 

In addition to IQ domain mutations in the cardiac Na_v_1.5 channel, there are several mutations in other domains of Na_v_1.5 involved in the regulation of CaM interaction with the sodium channel. In the N-terminal domain of the Na_v_1.5 channel, one Brugada syndrome mutation R121W was reported to weaken the interaction between CaM and the channel [54]. In the III-IV linker of the Na_v_1.5 channel, Brugada syndrome mutation K1493del (a Lys was deleted at the position 1493) decreased Ca^2+^-dependent CaM-interaction with Na_v_1.5 channel [83]. Brugada syndrome mutations Y1494N and I1521K were shown to cause domain-specific perturbations of the interaction with CaM [51]. In the EF hand-like (EFL) motifs upstream of the IQ domain of Na_v_1.5 channel, mutations (L1825P and Y1795insD) associated with LQT and Brugada syndromes perturbed CaM regulation of the channel function [84]. The persistent sodium currents produced by another Brugada syndrome mutation E1784K in the EFL motif were modulated by CaM overexpression [23]. Moreover, the mutation F1759A near the EFL motifs tuned the late sodium currents in CaM binding-deficient channels in cardiac myocytes [26].

### 5.4. Na_v_1.8 Mutations 

Na_v_1.8 channel IQ domain mutations were reported to be linked with cardiac arrhythmias. One clinical research reported Na_v_1.8-R1863Q mutation in a patient with Brugada syndrome (BrS) [85]. The Na_v_1.8 mutation R1869C was identified in an index case with atrial fibrillation (AF) and BrS [86]. In addition, mutation Na_v_1.8-R1869G was identified to co-segregate with familial AF [87]. 

All IQ domain mutations influence CaM interaction with the Na_v_1.8 channel. Na_v_1.8-R1863Q reduced the CaM-induced hyperpolarization shift of the voltage dependence of inactivation of the channel. Na_v_1.8-R1869C and Na_v_1.8-R1869G disrupted CaM-induced hyperpolarization shift and attenuated effects of CaM on development and recovery from slow inactivation [60]. These results suggested that Na_v_1.8 IQ domain mutations weakened the interaction between CaM and Na_v_1.8 channel and perturbed CaM regulation of Na_v_1.8 function.

## 6. Conclusions and Perspectives

We have described the interaction between CaM and each isoform of voltage-gated sodium channel family and summarized mutations associated with human diseases in CaM regulation of voltage-gated sodium channels.

The effects of CaM on the sodium channels are complex. They are not only involved in various regulation ways but also related to multiple factors in the cells. CaM regulates Na_v_ channel gating by binding to the IQ motif of the channel. Intracellular Ca^2+^ is also reported to modulate cardiac Na_v_1.5 channel inactivation by binding to an EF hand-like (EFL) motif of the sodium channel. The multiple binding sites of Ca^2+^ (either in the EFL of Na_v_ channel or in the EF-hand of CaM) and ability of CaM binding to the IQ domain of the Na_v_ channel result in various regulations of Ca^2+^ and/or CaM on the Na_v_ channel function. Therefore, delineating Na_v_ modulation by CaM will require developing novel approaches that exclude or minimize other factors’ modulation on CaM regulation of sodium channel function. In addition, studies have shown that CaM has isoform-specific modulation of voltage-gated sodium channels [31,32,33,34]. Because sodium channel isoforms have specific tissue distribution (Figure 1), whether environmental factors in different tissues are involved in CaM regulation of Na_v_ function remains unclear. Studies in the field will help explore the regulatory mechanism underlying distinct properties of CaM modulation of voltage-gated sodium channel function. Moreover, although the canonical binding site of CaM is the C-terminal domain of the sodium channel, a recent study showed another interaction site between CaM and the channel. A putative CaM-binding sequence comprising 26 amino acids is located at the N-terminal domain of the sodium channel [54]. Future studies on the complexes of a Na_v_ N-terminal domain and CaM will extend and provide a complete understanding of the contribution of CaM on the Na_v_ function.

In summary, CaM plays a critical regulatory role in cellular electrophysiology by its ability to bind to voltage-gated sodium channels. Understanding how it regulates sodium channel function is a crucial step towards developing treatments for diseases associated with sodium channel mutations.

## Figures and Tables

**Table 1:** CaM-binding IQ domain mutations in Na_v_ channels.

Pathological Mutation in CaM-Binding IQ Domain	Change in Nucleotide	Phenotype	Effect On Na_v_ Function
Na_v_1.1-I1922T	c.5765T>C	Dravet syndrome	unknown
Na_v_1.1-R1928G	c.5782C>G	Familial epilepsy syndrome Familial hemiplegic migraine Cryptogenic epileptic syndrome Dravet syndrome	unknown
Na_v_1.2-R1902C	c.5704C>T	Familial autism	(1) Abolished Ca^2+^-dependence of CaM binding; (2) Induced hyperpolarization shift of the voltage dependence of activation and inactivation of the channel.
Na_v_1.2-R1918H	c.5753G>A	Idiopathic generalized epilepsy	(1) Induced an increased late sodium current; (2) Overexpression of CaM reduced the late sodium current.
Na_v_1.5-R1898C	c.5692C>T	Brugada syndrome	unknown
Na_v_1.5-R1898H	c.5693G>A	Arrhythmogenic right ventricular dysplasia/cardiomyopathy	(1) Caused a reduction in peak sodium current; (2) Caused a structural deficit and decreased abundance of Na_v_1.5 and N-Cadherin clusters.
Na_v_1.5-E1901K	c.5701G>A	Brugada syndrome	unknown
Na_v_1.5-E1901Q	c.5701G>C	Long QT syndrome	(1) Induced an increased late sodium current; (2) Overexpression of CaM reduced the late sodium current.
Na_v_1.5-S1904L	c.5711C>T	Long QT syndrome Brugada syndrome	(1) Reduced binding affinity between channel and CaM; (2) Induced an increased late sodium current.
Na_v_1.5-Q1909R	c.5726A>G	Long QT syndrome Sudden infant death syndrome	(1) Induced an increased late sodium current; (2) Overexpression of CaM reduced the late sodium current.
Na_v_1.5-R1913H	c.5738G>A	Long QT syndrome	(1) Induced an increased late sodium current; (2) Overexpression of CaM reduced the late sodium current.
Na_v_1.5-R1919C	c.5755C>T	Long QT syndrome Brugada syndrome	unknown
Na_v_1.5-A1924T	c.5770G>A	Brugada syndrome	(1) Induced a hyperpolarization shift in voltage-state activation; (2) Generated a reduced slow inactivation that was rescued by CaM.
Na_v_1.8-R1863Q	c.5588 G>A	Brugada syndrome	(1) Reduced CaM-induced hyperpolarization shift of the voltage dependence of inactivation of the channel.
Na_v_1.8-R1869C	c.5605 C>T	Brugada syndrome	(1) Disrupted CaM-induced hyperpolarization shift; (2) Attenuated effects of CaM on development and recovery from slow inactivation.
Na_v_1.8-R1869G	c.5605 C>G	Atrial fibrillation	(1) Disrupted CaM-induced hyperpolarization shift; (2) Attenuated effects of CaM on development and recovery from slow inactivation.

## Abbreviations

Na_v_	Voltage-gated sodium channel
CaM	Calmodulin
Apo-CaM	Ca^2+^-free CaM
Ca^2+^/CaM	Ca^2+^-binding CaM
IQ domain	A domain with highly conserved residues isoleucine (I) and glutamine (Q)
CBP	Calmodulin-binding peptide
CIP	Calmodulin inhibitory peptide
EFL	EF hand-like motifs
FHF	Fibroblast growth factor homologous factor
FGF	Fibroblast growth factor
CaMKII	Calcium/calmodulin-dependent protein kinase II
AP	Action potential
DRG	Dorsal root ganglia
SCG	Superior cervical ganglion
NMR	Nuclear magnetic resonance
GEFS+	Generalized epilepsy with febrile seizures plus
FHM	Familial hemiplegic migraine
SMEI	Severe myoclonic epilepsy of infancy
SIDS	Sudden infant death syndrome
LQT3	Type 3 of long-QT syndrome
